# Effect of warm/cool white lights on visual perception and mood in warm/cool color environments

**DOI:** 10.17179/excli2021-3974

**Published:** 2021-08-31

**Authors:** Reza Shahidi, Rostam Golmohammadi, Mohammad Babamiri, Javad Faradmal, Mohsen Aliabadi

**Affiliations:** 1 Center of Excellence for Occupational Health, School of Public Health and Research Center for Health Sciences, Hamadan University of Medical Sciences, Hamadan, Iran; 2 Social Determinants of Health Research Center, Department of Ergonomics, School of Public Health, Hamadan University of Medical Sciences, Hamadan, Iran; 3 Modeling of Noncommunicable Diseases Research Center & Department of Biostatistics and Epidemiology, School of Public Health, Hamadan University of Medical Sciences, Hamadan, Iran; 4 Center of Excellence for Occupational Health, Occupational Health and Safety Research Center, School of Public Health, Hamadan University of Medical Sciences, Hamadan, Iran

**Keywords:** lighting, color, correlated color temperature, visual perception, mood

## Abstract

Color and light are two ambient attributes for interior spaces that can be used in the design and modification of workspaces. The visual and psychological effects of color and light of each have been studied separately and widely. The aim of this study was to investigate the simultaneous effects of warm/cool white light on visual perception and mood in a simulated colored workspace. Thirty-three healthy male participants were recruited. They were asked to judge the visual perception and mood of three types of workspace that were designed by colors of white, red, blue, and lights of a cool and warm white in the random six sessions. The participants have experienced higher levels of tension, anger, depression, anxiety and lower levels of visual comfort, attractiveness, brightness and calmness of environment in the red condition than to white in both state of light. The blue wall reduced brightness and increased attractiveness of environment compared to white wall. Cool white light reduced the warmth of color and increased brightness in all three color environments compared to warm light. The preference for cool or warm light depends on the color of the environment's indoor surface. It seems that the combination of the white color and warm light or the blue color with cool light has a more favorable effect on visual perception and people's mood in workplaces.

## Introduction

Color and light are ambient attributes that can be used in the design and modification of interior spaces for improving visual perception, mood and performance (Savavibool, 2016[21]; Chao et al., 2020[4]). Various researches have shown the same effect of light and color on the activity of parts of the brain (amygdala and hypothalamus) that are involved in emotional and mood responses (Sokolova and Fernández-Caballero, 2015[25]; Fechir et al., 2010[9]; Rautkylä et al., 2012[19]). Color has three characteristics of hue, brightness, and degree of saturation, each of which has different effects on the autonomic nervous system and the physiological and psychological responses (AL‐Ayash et al., 2016[1]; Sorokowski and Wrembel, 2014[26]; Wilms and Oberfeld, 2018[31]). People prefer different colors depending on the circumstance (Jalil et al., 2012[11]). From a psychological point of view, some colors have a calming and concentrating effect (e.g. blue), others are stimulating (e.g. red) and still others are anxiety-inducing (Elliot et al., 2007[8]; Wilms and Oberfeld, 2018[31]; Kurt and Osueke, 2014[15]). Blue is the most popular color, because it is cool, comfortable, relaxing, peaceful, and calming. Red is considered warm and arousing and stimulates human feelings and activates people. Moreover, white color is considered more ineffective and somewhat boring. Colors also affect visual perception (Yildirim et al., 2015[33]; Savavibool and Moorapun, 2017[22]). As a result, colors can also affect the mood. However, some studies show that there is no significant difference in the mood of people in exposure to different colors (von Castell et al., 2018[28]). Researchers believe that the reason for this disparity is individual differences (such as personality, age, gender, culture, experience, and momentary feelings) (Dijkstra et al., 2008[6]; Baniani and Yamamoto, 2015[2]). The quantity and quality of lighting is another ambient factor that can also cause a wide range of non-visual response (Westland et al., 2017[30]; Ru et al., 2019[20]). Lighting can affect optimal visibility, mood, behavior, alertness, and cognitive performance (te Kulve et al., 2018[27]; Correa et al., 2016[5]). The mechanism of light-induced non-visual responses is attributed to the neural association of light receptors called intrinsically photosensitive retinal ganglion cells (IPRGCs) with the suprachiasmatic nucleus (SCN), in which the blue spectrum and intensity play a major role (Ru et al., 2019[20]). However, many non-visual phenomena of light, especially during daylight hours, cannot be fully explained by this mechanism. Thus, the possible role of rod and cone photoreceptors in this mechanism has been suggested (Lucas et al., 2012[16]). One of the important descriptors of lighting quality is the correlated color temperature (CCT). In general, studies that have been conducted on the effect of CCT have shown that the high CCT (Cool lights) decreases the positive mood compared to the low CCT (Warm lights) with equal illuminance (Smolders and de Kort, 2017[24]). The change in color temperature affects the visual appraisal of the environment (Manav, 2007[17]; Kazemi et al., 2018[12]). It can be hypothesized that CCT by changing three characteristics of color can affect the visual perception and mood of people due to the change in the spectral reflectance spectrum caused by the coefficient of spectral reflection of the surfaces. Therefore, the effect of color and light should be studied simultaneously. Also, in terms of building design, light and color are interdependent and should be considered as part of a more complex system for studying human mood (Küller et al., 2006[13]). However, in the studies of color, the effect of light sources has not been considered, and only in some cases the color temperature has been mentioned. Also, most studies do not mention the color of the surfaces in the subjects' field of view, except for neutral color, despite the commonalities between color and light in the reflective spectrum. Since the colors and light sources are used in workspaces with different characteristics, comparing the results from such experimental studies and generalizing to real environments is not entirely accurate. This study aimed to investigate the combined effects of surfaces with white, red and blue colors respectively as neutral, warm and cool and the 3000K and 6500K LED light sources respectively as warm and cool white lighting on visual perception and mood in a simulated workspace.

## Methods

### Participants 

The study was an experimental study. The participants were 33 healthy males by 24.66 ± 3.29 years' old (range 21-35 years old). The inclusion criteria were: Lack of definite evening or morning chronotype according to MEQ questionnaire, no eye diseases, and color blindness according to Ishihara test, no neurological, cardiovascular, autoimmune, and pulmonary diseases, non-smoking, non-alcohol and non-drugs, good general health (final score less than 22 according to the GHQ questionnaire), no coffee consumption a week before the test, enough and good quality sleep, and waking up at least one hour before the test. For this aim, each participant completed the questionnaires of the general health (GHQ) and Morning-Evening questionnaire (MEQ) to determine the chronotype. Moreover, the Ichihara test was performed to determine color blindness. The level of participants' anxiety and their personality traits were measured using the Spielberger State-Trait Anxiety Inventory (STAI) questionnaire and the Michel Gauquelin questionnaire, respectively. The STAI is a commonly used measure of trait and state anxiety. It has 20 items for assessing trait anxiety and 20 for state anxiety. Each item is rated on a 4-point Likert-type scale (e.g., from “Almost Never” to “Almost Always”) and given values from 1 to 4. Higher total scores indicate greater anxiety. The Michel Gauquelin Questionnaire has 50 two-choice questions that score on two subscales of introversion and extroversion. In a way, the sum of a person's introversion and extroversion scores is equal to 50. In an ambivert person, both introversion and extroversion scores are equal to 25. It should be noted that in this study, the introversion score of individuals has been analyzed. That is, the higher the number, the greater the tendency towards introversion. Finally, the consent form was signed by all participants, and the Medical Ethics Board at the Hamadan University of Medical Science, Iran approved this study. 

### Study design and experimental room 

Two rooms were designed in the research laboratory of the School of Public Health, Hamadan University of Medical Sciences. Room number 1 (dimensions: 7×7×3 m) was designed by the usual office equipment with cream-colored walls. The CCT and the illuminance level of the chamber were 4000K, and 500 lux respectively. This room was designed for the preparation and measuring of the baseline variables of participants.

Room number 2 was the experimental room (dimensions: 8×7.5×2.5m). This room was partitioned into three booths with dimensions of 2.7×2.5×2.5 m. Each booth was designed similar to an office. The walls of booths number 1, 2, and 3 were covered with wood panels with straight sides in colors of red, white, and blue, respectively. In order to avoid glare effects, also the floor of the room was covered with brown carpet. The lighting was provided by 6 LED panel light 12 watt and 4 LED linear light 2 watt, some of which were lit to keep the illuminance the same in different designs of the booths. The lights were installed higher than the height of the partitions so that they were not in direct view of the participants. One desk was located in the center of the room with a 19 inch monitor on it. The illuminance level of the booths was 500 Lux, according to ISO 8995:2001, which is recommended for offices and control rooms. The room was shielded from the daylight. The equivalent background noise level was less than 45 dB(A). Airflow velocity, ambient temperature, and relative humidity were controlled at 0.15 m/s, 25 °C, and 50 %, respectively, during the experiments. The color specifications of the partitions (measured by the Xrite SP-64 spectrophotometer) are presented in Table 1(Tab. 1). The overall reflectance of the white, blue and red partitions were 0.82, 0.16 and 0.17 respectively.

**Figure 1:**
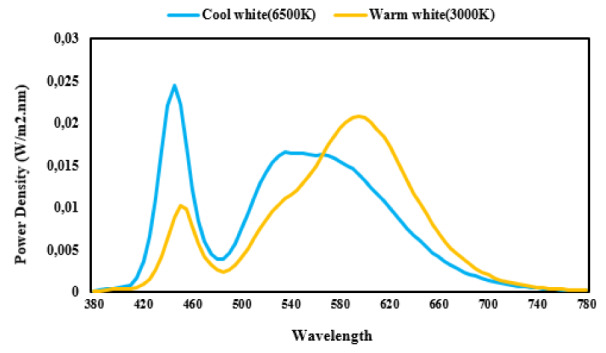
Spectral power distribution curve of two LED lamps

**Figure 2:**
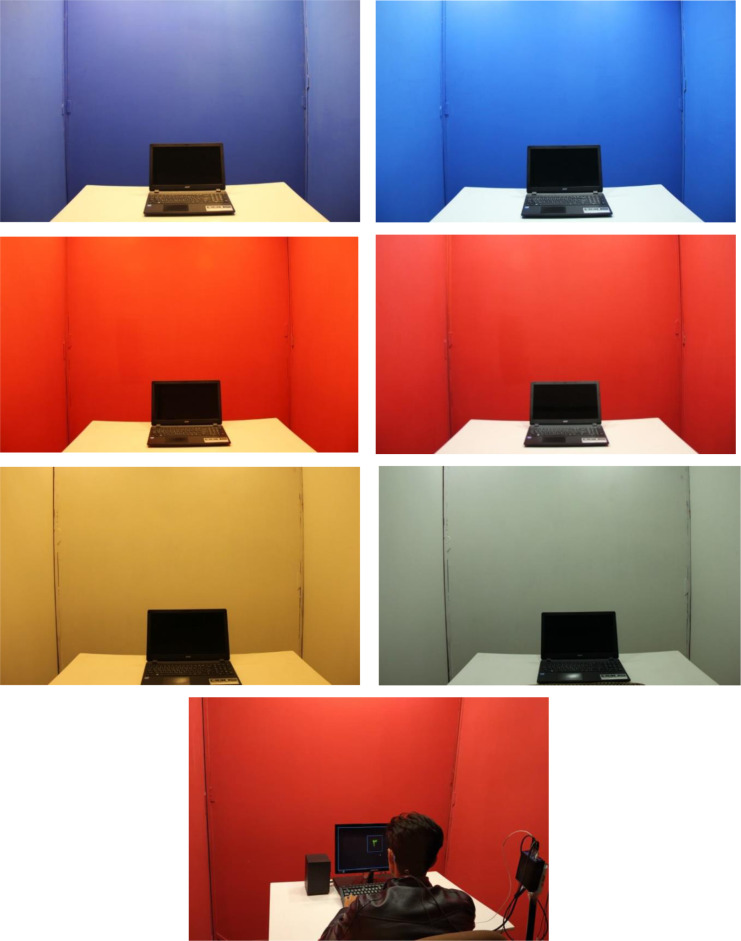
Experimental setup and six conditions' design. The photos on the left and the right are taken under warm white and cool white light respectively.

The Sekonic Spectromaster C7000 spectrometer was used to characterize the light of sources, on the desk and at different angles of the field of view. The curve of the spectral power distribution of the two types of LEDs is shown in Figure 1(Fig. 1). Color rendering index (CRI) of warm white LED and cool white LED was 82.5 and 80.4 respectively. The experimental setup is shown in Figure 2(Fig. 2). Since the direction of vision of the participants during the experiment was in the direction facing and on the desk chiefly, then the light values were measured in the vertical direction and at a 45 degree downward angle at eye level. Table 2(Tab. 2) shows the photometric values in these two directions. The CIE α-opic toolbox was used to calculate the photometric values from spectral data (Schlangen and Price, 2021[23])*.*

### Experiment procedure

To investigate the combined effects of two CCT; 3000 vs. 6500K (warm white vs. cool white) and the color of walls, a workspace was simulated in three different colors of white, blue, and red. Therefore, the experiments were designed in six sessions. The sessions were conducted randomly on six consecutive days in the morning and each session lasted for 130 minutes. At first, a training session about the tests was held to become familiar participants with tests. Before the participants entered the experimental room, the level of anxiety and the mood status using the State-Trait Anxiety Inventory (STAI) and Brunel Mood Scale (BRUMS) was determined in room number 1. Brunel Mood Scale assesses tension, anger, depression, calmness, confusion, vitality, fatigue, and happiness components. 

**Figure 3:**
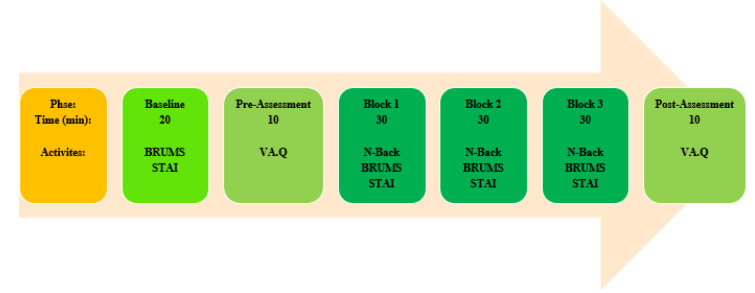
Outline of the experiment

These data were recorded as the baseline. In the next phase, the participant entered the main test room and after 5 minutes, the subject completed the visual assessment questionnaire. This questionnaire was used to assess visual perception components of environment. Then, the main test was conducted in three-time block 30 minutes. To impose mental workload at different levels in each block, the subjects were asked to do the N-Back task as a cognitive test at one of three levels (1 to 3) which had been counterbalanced in the three blocks mentioned according to the experimental design. After doing cognitive tests in each of the three blocks, Brunel Mood Scale and STAI were again completed. In post-assessment phase, the visual assessment of the environment was conducted. Throughout the experiment, the illuminance and CCT were monitored using a spectrometer. Figure 3(Fig. 3) illustrates the process of performing experiments and tests during each session. 

### Data analysis

Descriptive analysis was performed based on mean ± SD for quantitative variables. In this study due to the continuous measurements of the data, the linear mixed-effects model (LMM) was used. Therefore, an LMM model was developed in software R version 4.0.3 to estimate the effects of the color (red, blue, and white) and CCT (warm and cool light) on 8 components of mood, state anxiety and 9 subscales of visual assessment and moderating confounding variables (quantity of the variables that were measured at the baseline phase, anxiety trait, personality trait and N-Back levels). Based on determined predictive variables involved in the LMM model by software R, SPSS version 26 software was used to show EMM bar graphs related to changes in mood and anxiety variables than to the baseline. 

## Results

The average GHQ score of the subjects was 15.45 ± 3.96. Moreover, the average anxiety trait and PSQI scores of subjects were 36.59 ± 7.77, and 4.6 ± 2.7, respectively. The averages extraversion score of the participants obtained according to Michel Gauquelin's questionnaire was 22.6 ± 6.7. Moreover, the averages introversion score was 27.4 ±. 6.7. None of the participants was extremely evening or morning type.

### Anxiety and mood

Table 3(Tab. 3) presents the descriptive statistics of mood state and anxiety variables in the six simulated conditions. 

**Figure 4:**
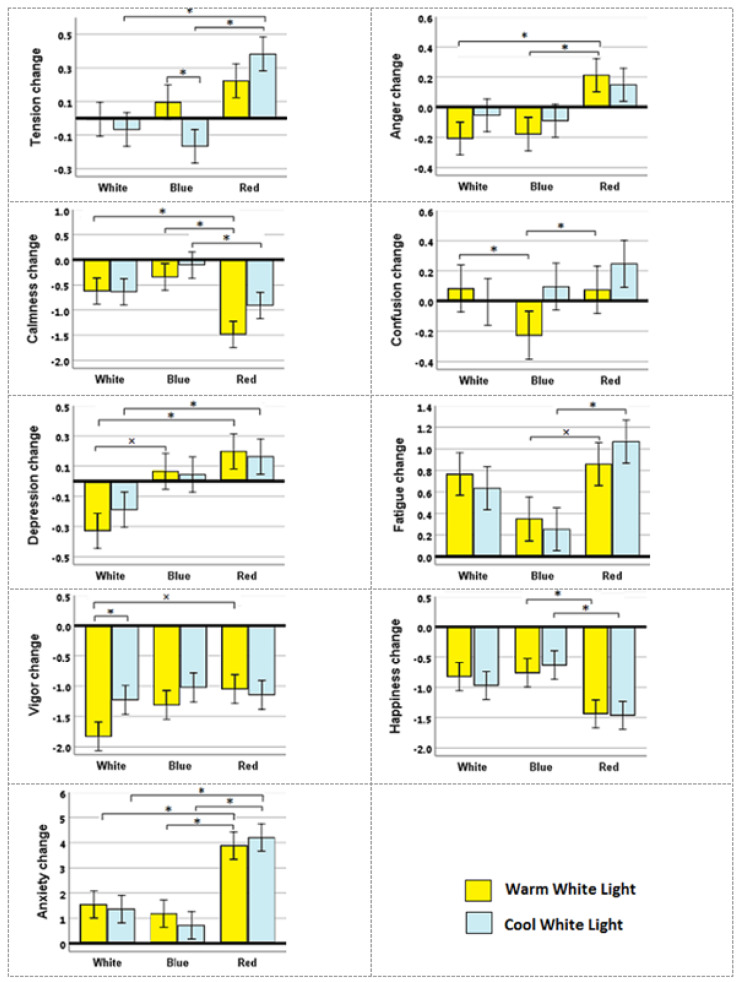
Changes from baseline in individual mood and anxiety variables using the LMM model. * stands for p <0.05 and × for p <0.1.

Moreover, the changes of each variable of mood and anxiety than to the baseline are presented in Figures 4(Fig. 4). The results of correlation analysis of six simulated conditions for the mood and anxiety variables using the LMM model are also summarized in Table 4(Tab. 4). 

As shown in Figure 4(Fig. 4), the participants have experienced a higher tension level in the red condition than to white and blue condition, especially at cool light. Whereas, there were no significant differences in tension level between blue and white conditions. The tension level was consistent in white conditions with warm light compared to the baseline state, whereas, it somewhat increased in blue or red conditions and warm light. However, these differences were not significant. The use of cool light in white and blue environments decreased the tension level compared to the baseline, which was more evident in blue and caused a significant difference in warm light. The red condition in the presence of cool light significantly increased the tension level compared to the white and blue conditions. 

The red color increased the anger level more than the white or blue color. The anger level decreased during exposure to white and blue color conditions and warm white compared to baseline. The red color of the environment increased the rate of anger significantly in presence of lighting by warm white or cool white. However, the blue and white colors in presence of warm light somewhat reduced the rate of anger more than cool light. The red color reduced the calmness in both cool and warm light compared to white and blue and the reduction rate of calmness in blue color was less than others. Concerning the combined effects of color wall and light on the confusion, the results showed that the blue color in the presence of warm light reduced confusion and had a significant difference. Whereas, the red color under the cool white light caused the most confusion during the experiment. Moreover, the level of calmness reduced by increasing exposure time in all conditions compared to baseline. White color reduced depression. The red color more than blue and the blue color more than white has caused the depression. The fatigue rate was higher for red than for white and blue, but the differences were significant only compared to blue for both types of light. Also, cool light in white and blue colors caused less fatigue than warm light, while in red color this trend was reversed. However, the results were not significant. 

According to Table 4(Tab. 4), the vigor feeling of individuals was gradually reduced during the experiments in all sessions. This decrease was greatest in white color with warm light. Warm light in a white color environment significantly reduced the amount of vigor than cool light. The decreasing effect of red color on vigor was less than white color in warm light. The decreasing effect of blue color on the level of happiness was less than others, especially compared to red. The effect of light on the amount of happiness was not significant in different colored environments. 

The anxiety level increased than the baseline in all sessions. This increase was higher in red color than white, while it was lower in blue color compared to white. The red color increased the anxiety level significantly compared to the other colors in the presence of both warm and cool light.

According to Table 4(Tab. 4), subjects with higher anxiety trait experienced more tension, depression and fatigue and less calmness, vigor and happiness. As time went on, participants fatigue and state anxiety levels increased and confusion, calmness, vigor and happiness decreased. Also, with increasing exposure time to cool light, an increasing rate of anger and depression appeared. Subjects with higher introversion scores had more anger, depression and state anxiety. Introverts had more anxiety. The result of model fitting showed that the different levels of the N-Back task had no significant effect on the measured variables of anxiety and mood.

### Visual perception

The visual assessment questionnaire evaluates 9 components of the environment, including visual comfort, attractiveness, spaciousness, calming effect, peacefulness, exciting effect, pleasuring, warmth of color, and brightness. The results of the descriptive statistics of visual perception variables in different color and light conditions were presented in Table 5(Tab. 5). Moreover, the results of correlation analysis of six simulated conditions for the visual perception variables using the LMM model have been summarized in Table 6(Tab. 6).

According to Table 6(Tab. 6), the level of visual comfort was slightly higher in blue color than white but significantly lower in red color. Anxious people had less visual comfort. The level of perceived brightness diminished in the blue and red colors compared to white. The cool white environment seemed to be brighter than warm white. The attractiveness of blue color was more than white and red colors, and red color was less than white. The type of light had no significant effect on the attractiveness of the environment. The participants described the blue color as cooler than the white, and the red color warmer. The warmth of color in cool white was less than warm white. The warmth of color had the lowest value in blue and the highest in white.

The light and color had no significant effect on the perception of the size of the room (Roomy), and only anxious people described the space as smaller. Furthermore, regarding the environment calming, the participants reported that white color is calmer than red. However, there was no significant difference in the blue color. Also, cool light reduced the calming score of the environment compared to warm light. In general, the score of environment calming was low in the anxious people. In general, the white color was evaluated as peacefulness color. However, the effect of light and color on peacefulness was not significant. Participants described the red and blue colors as more exciting than white, respectively. Moreover, the warm light has been described as more exciting than the cool light. Over time, the environment exciting was diminished, and anxious people described the environment as lower exciting. The pleasuring degree of the red color was significantly less than the other two colors. Moreover, the type of light did not affect this component. Generally, the level of pleasuring of the environment was lower in anxious people. Different levels of N-Back task didn't have any significant effect on the measured variables of visual perception.

## Discussion

This study examines the effect of warm/cool white lights of LED lamps on visual perception and mood in simulated color environments. Designing a favorite work environment or office buildings in term of psychology, ergonomics and engineering is a subject that less has been studied (Bluyssen et al., 2011[3]). In addition to prospective, spaciousness and other technical and environmental parameters (i.e. thermal factors, moisture, noise and vibration, radiation, chemical compounds, particulates), the indoor building conditions in particular color and lighting aspects are two critical factors that with the aim of wellbeing (health and comfort) should be taken into consideration (Bluyssen et al., 2011[3]; Manav, 2007[17]). In this regard, considering individual characteristics is also very important in design (Elliot et al., 2007[8]; Küller et al., 2006[13]). In general, introverts as well as anxious people were more affected by unfavorable environmental conditions. In other words, introverts scored lower in the positive components of mood and higher in the negative components. Anxious people scored lower on the positive components of visual assessment. The reason for this can be seen in the different activity of the autonomic nervous system in people with different personality traits (Küller et al., 2009[14]; Wilms and Oberfeld, 2018[31]; von Castell et al., 2018[28]). Therefore, in the design of light and color in indoor spaces, it is necessary to consider the personality traits of individuals. 

The results of the current study also indicated that color as a strong factor affects mood and anxiety. This issue was more severe for red color and increased tension, anger, depression, fatigue and anxiety and decreased people's calmness and happiness. In addition, increased levels of anxiety can be due to the stress effect of the red color. In this regard, Elliot and colleagues (2007[8]) and von Castell and colleagues (2018[28]) have also reported that the red color generally causes a negative mood in people. The results of these studies were consistent with the results of the current study. Scores on visual environmental variables for all three colors were low. However, the blue color score was more desirable. The pleasuring rate of the colors blue and white were equal to and greater than red. The calming and exciting effects of red and blue colors were relatively similar and had a more exciting effect. In general, the effect intensity of the red color was more. The red color also was more exciting, unpleasant, and unsafe than the other two colors. Moreover, this color reduces visual comfort and increases the temperature of the perceptual color. Comparison of visual assessment of blue color with white showed that there was no significant difference in many of these cases. In general, the blue color compared to the red reduced the level of state anxiety, anger, fatigue, and confusion and a relative increase in happiness, peacefulness, and vigor. In this regard, the results of our study are consistent with many previous studies (Ekström and Beaven, 2014[7]). In an experimental study, Küller and colleagues (2009[14]) reported that a good color design will improve the overall mood and well-being of people. These results were consistent with the current study. Concerning the effect of light type on the mood and visual assessment, the results also showed that the cool white light causes to appear brighter in white color the indoor environment. On the other hand, the confusion and depression under warm light were less and more for blue color than white, respectively. In this regard, in a field study, Wei and colleagues (2014[29]) studied the effects of CCT and lighting on the visual comfort and brightness perception of twenty-six subjects. They reported that the combination of higher CCT with lighting causes higher brightness. The combination of higher CCT with lighting causes higher brightness and participants gave lower ratings for visual comfort and color temperature. They also perceived the environment as cool (Wei et al., 2014[29]). Whereas, Manav and colleagues (2010[18]) reported that the light yellow room is more “stimulating” than the light blue room while the mean of the responses is the same under both of the rooms for the impression of “intimate”. Cool white light reduced the effect of feeling the warmth of different colors. Cool light compared to warm light increased vigor in white color and decreased fatigue in blue and white. However, the effect of light on other components was not significant. In this regard, the previous studies also have reported that the CCT alone is not effective on visual comfort. In fact, the desirable design depends on a balance between the color of the interior surfaces and the CCT (Yang and Jeon, 2020[32]; Sokolova and Fernández-Caballero, 2015[25]; Huang et al., 2020[10]). Therefore, it seems that white or warm light is less effective during the day and especially in the morning.

In general, the results showed that over time, participants' descriptive assessments of the light and color in the indoor environments shifted toward equilibrium. This effect can be attributed to the gradual adaptation of people to the environment. Hence, time is a key factor in the study of the effect of light and color on visual perception. Because a different effect may result from increasing the duration of presence in an indoor environment with specific light and color.

During this research, some limitations should be mentioned. The participants were all male and the female were not studied. The number and duration of test sessions were relatively large. Another obstacle was that the mental state of the subjects was uncontrollable before entering the test session. Although participants had to follow the experiment schedule. However, it was not possible to fully control the nutritional status of individuals.

## Conclusion

The results showed that color as a strong factor affects many parameters of visual perception, mood, and anxiety. Correlated color temperatures also have some effect on these variables. However, the preference for cool or warm light depends on the color of the environment's indoor surface. In total, it seems that the combination of the white color and warm light or the blue color with cool light has a more favorable effect on visual comfort and people's mood in workplaces.

Moreover, regardless of environmental parameters, the personality trait and anxiety level are the individual factors that have the main role on mood and visual perception. Therefore, in visual designing of indoor surfaces of buildings and workspaces can be used the blue or white color and even red in combination with cool or warm light considering individual and personality factors.

## Declaration of competing interest

The authors declare that they have no known competing financial interests or personal relationships that could have appeared to influence the work reported in this paper. 

## Acknowledgements

The authors thank the students who participated in the study as well as Hamadan University of Medical Sciences – Iran (under project No. 113192) for their support of the research.

## Figures and Tables

**Table 1:**
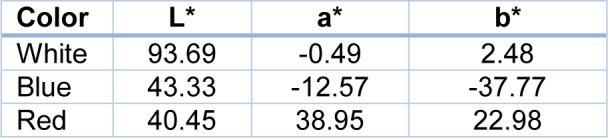
Values of the color coordinates of the partitions sample in CIELAB system under D65 light

**Table 2:**
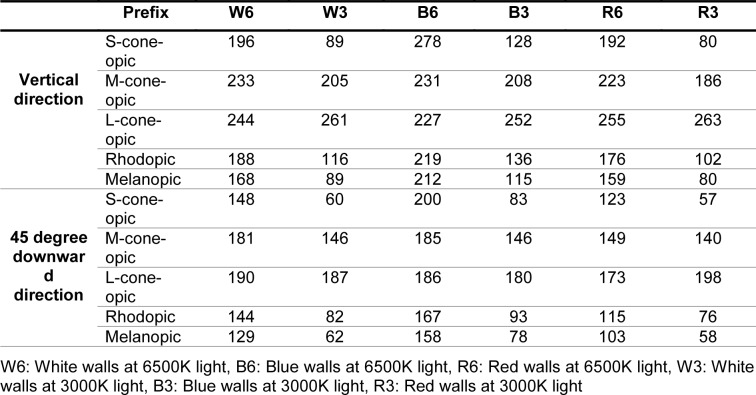
Photometric values measured in the vertical direction and 45 degrees downwards at eye level (α-opic equivalent daylight (D65) illuminance, lx)

**Table 3:**
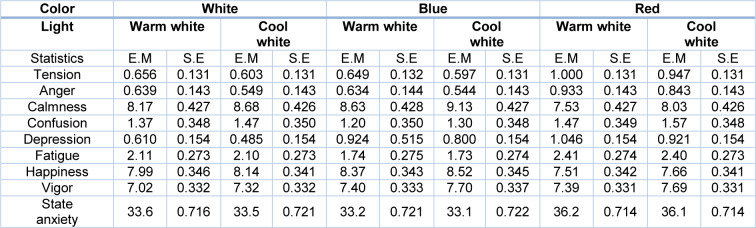
Estimated marginal means for mood state and anxiety variables

**Table 4:**
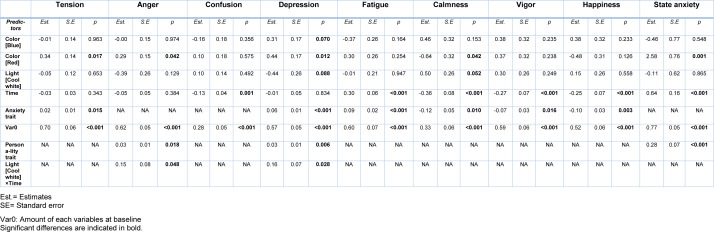
Results of correlation analysis of six simulated conditions for the mood and anxiety variables using the LMM model

**Table 5:**
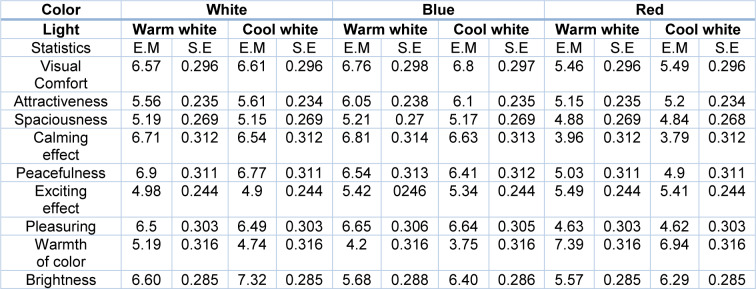
Estimated marginal means for visual perception variables

**Table 6:**
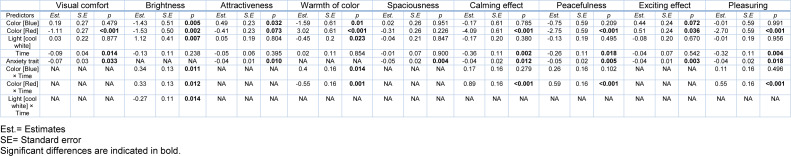
Results of correlation analysis of six simulated conditions for the visual perception variables using the LMM model
